# NeuroMath: Advanced Methods for the Estimation of Human Brain Activity and Connectivity

**DOI:** 10.1155/2009/275638

**Published:** 2009-10-19

**Authors:** Laura Astolfi, Andrzej Cichocki, Fabio Babiloni

**Affiliations:** ^1^Department of Informatic and Systems, Faculty of Engineering, University of Rome “Sapienza”, Via Ariosto 25, 00185 Rome, Italy; ^2^Laboratory for Advanced Brain Signal Processing (ABSP), RIKEN Brain Science Institute, 2-1 Hirosawa, Wako-shi, Saitama 351-0198, Japan; ^3^Department of Physiology and Pharmacology, School of Medicine, University of Rome “Sapienza”, 00185 Rome, Italy

It is well known that the electroencephalography (EEG) and magnetoencephalography (MEG) are useful techniques for the study of brain dynamics, due to their high temporal resolution. However, it is known that the spatial resolution of the standard EEG is rather low, due to the different electrical conductivities of brain, skull, and scalp that markedly blur the EEG potential distributions, making the localization of the underlying cortical generators problematic. In the last ten years, a body of mathematical techniques, known as high-resolution EEG, was developed to estimate more precisely the cortical activity from non-invasive EEG measurements. Such techniques include the use of a large number of scalp electrodes, realistic models of the head derived from magnetic resonance images (MRIs), and advanced processing methodologies related to the solution of the so-called “inverse problem,” that is, the estimation of the brain activity (i.e., electromagnetic generators) from the EEG/MEG measurements. The approach implies both the use of thousands of equivalent current dipoles as a source model and the realistic head models, reconstructed from magnetic resonance images, as the volume conductor medium. The use of geometrical constraints on the position of the neural source or sources within the head model generally reduces the solution space (i.e., the set of all possible combinations of the cortical dipoles strengths). An additional constraint is to force the dipoles to explain the recorded data with a minimum or a low amount of energy (minimum-norm solutions). The solution space can be further reduced by using information derived from hemodynamic measures (i.e., fMRI-BOLD phenomena) recorded during the same task. The rationale of a multimodal approach is that neural activity, modulating neuronal firing and generating EEG/MEG potentials, increases glucose and oxygen demands. This results in an increase in the local hemodynamic response that can be measured by functional magnetic resonance images (fMRIs). Hence, fMRI responses and cortical sources of EEG/MEG data can be spatially related, and the fMRI information can be used as a priori in the solution of the inverse problem. As a result of all these computational approaches, it is possible to estimate the cortical activity with a spatial resolution of few millimeters and with a temporal resolution of milliseconds from non-invasive EEG measurements.

 However, static images of brain regions activated during particular tasks do not convey the information of how these regions communicate to each other. The concept of brain connectivity is viewed as central for the understanding of the organized behavior of cortical regions beyond the simple mapping of their activity. This organization is thought to be based on the interaction between different and differently specialized cortical sites. Cortical connectivity estimation aims at describing these interactions as connectivity patterns which hold the direction and strength of the information flow between cortical areas. To achieve this, several computational methods have been already applied on data gathered from both hemodynamic and electromagnetic techniques. By using such methods, it is possible to infer the information flows between the cortical areas in human during particular motor and cognitive tasks. Possible applications of this promising technology are in the fields of the study of human behavior and cognition and in the brain computer interface area. Methods for the estimation of brain connectivity have been scattered proposed in literature related to the fMRI, NIRS, and EEG or MEG technologies.

 Hence, the main objective of this special issue is to increase the knowledge on the mathematical methods able to estimate the cortical activity and connectivity in the human brain from non-invasive neuroelectric and hemodynamic measurements. Such special issue includes both the state-of-technique review articles on the EEG and MEG methodologies written by world-class scientists, as well as the description of particular cortical models that can be used to generate cortico-cortical connectivity.

 Several papers of this special issue are devoted to the theme of brain-machine interfaces, treating the collection and the analysis of brain signals related to the imagination of motor acts, in the context of brain-computer interface. Applications of the techniques of source estimations were provided also in the field of neuroeconomics, with an example of the track of the brain activity with the EEG during the observation of TV clips, or even during the car driving, by using NIRS device, or in the study of emotional processing with the ERD/ERS techniques. Other papers in this special issue are related to the use of different advanced methodologies for the analysis of brain signals in psychiatric patients, and one is devoted to the description of a possible WEB structure for data sharing in the field of neuroscience.

 In summary, this special issue conveys interesting information about the state-of-the-art methodologies able to track the brain activity and connectivity during different tasks in the healthy subjects as well as in the psychiatric patients. We hope that the readership of CIN could appreciate this special issue as we appreciated it during its composition.

*Laura Astolfi* *Andrzej Cichocki* *Fabio Babiloni*

